# Supplementary material for: OpenAWSEM with Open3SPN2: A fast, flexible, and accessible framework for large-scale coarse-grained biomolecular simulations
Source: PLoS Comput Biol. 2021 Feb 12;17(2):e1008308. doi: 10.1371/journal.pcbi.1008308 (PMC7906472; doi:10.1371/journal.pcbi.1008308)
Supplement: S1 Document — Detailed description of the various term in openAWSEM and Open3SPN2 models along with all the parameter values. (PDF) [file pcbi.1008308.s001.pdf]

Supporting Information
OpenAWSEM with Open3SPN2: a fast, flexible, and accessible framework for large scale coarse-grained biomolecular simulations

Contents

1 OpenAWSEM **1**
- 1.1 Connectivity term 2
- 1.2 Chain term 2
- 1.3 Chirality term 2
- 1.4 Rama term 2
- 1.5 Excluded Volume term 3
- 1.6 Contact term 4
- 1.7 β-hydrogen bonding and P-AP terms 4

2 Open3SPN2 **5**
- 2.1 Bonded terms 7
- 2.2 Stacking, BasePairing and CrossStacking terms 13
- 2.3 Non-bonded terms 18
- 2.4 Protein-DNA Excluded Volume term 19
- 2.5 Protein-DNA Electrostatics term 19

3 Energy validation of the OpenMM implementation of AWSEM, 3SPN.2, and 3SPN.2C **20**
- 3.1 Energy evaluation comparison with LAMMPS AWSEM 20
- 3.2 Energy evaluation comparison with LAMMPS 3SPN.2 and 3SPN.2C 20

4 Tutorial **21**
- 4.1 open3SPN2 21
  - 4.1.1 Example DNA system 21
  - 4.1.2 Example Protein-DNA system 22

5 Supplementary figures **26**
- 5.1 Structure prediction results using three contact potential schemes evaluated using the overall Q 26
- 5.2 Example of over saturation of disulfide bonds observed in original AWSEM simulation. 26
- 5.3 Bets Q for each run. 26
- 5.4 The predicted structure of alpha-thrombin(PDB: 1ppb) aligned with the crystal structure. 28
- 5.5 The predicted structure of ribonuclease A(PDB: 1fs3) aligned with the crystal structure. 28

1 OpenAWSEM

In AWSEM coarse grained simulations, the amino acids are represented by six particles, (CA, CB, O, C, N and H) except for Proline and Glycine both of which are represented by five particles. For Proline, no hydrogen is connected to the

nitrogen inside its amide group. Glycine has no CB. Among those 6 particles in the standard representation, C, N and H are designated as "virtual sites" which means their coordinates are not dynamical variables but instead are computed based on the positions of the other particles, which are dynamical variables.

The standard AWSEM potential is made up of several term:

$$V_{AWSEM} = V_{con} + V_{chain} + V_{chi} + V_{rama} + V_{excl} + V_{contact} + V_{beta} + V_{pap} + V_{frag} \tag{1}$$

1.1 Connectivity term

The connectivity term is designed to maintain the bonded distances between $C\alpha_i$ and O_i, $C\beta_i$ and $C\alpha_{i+1}$. and between O_i to $C\alpha_{i+1}$.

$$V_{con} = k_{con}(\sum_{i}^{N}(r_{C\alpha_i O_i} - r^0_{C\alpha O})^2 + \sum_{res_i != GLY}(r_{C\alpha_i C_{\beta_i}} - r^0_{C\alpha\beta})^2 \tag{2}$$

$$+ \sum_{i}^{N-1}((r_{C\alpha_i C\alpha_{i+1}} - r^0_{C\alpha C\alpha_{i+1}})^2 + (r_{O_i C\alpha_{i+1}} - r^0_{OC\alpha_{i+1}})^2)) \tag{3}$$

1.2 Chain term

The chain term models the positions of C' and N atoms.

$$V_{chain} = \lambda_{chain}[\sum_{i=2}^{N}(\boldsymbol{r}_{N_i C_{\beta_i}} - \boldsymbol{r}^0_{N_i C_{\beta_i}})^2 + \sum_{i=1}^{N-1}(\boldsymbol{r}_{C'_i C_{\beta_i}} - \boldsymbol{r}^0_{C'_i C_{\beta_i}})^2 + \sum_{i=2}^{N-1}(\boldsymbol{r}_{N_i C'} - \boldsymbol{r}^0_{NC'})^2] \tag{4}$$

We implemented the connectivity term and the chain term using "HarmonicBondForce".

1.3 Chirality term

The chirality term is used to fix the direction of the C_{β_i} relative to the plane formed by C'_i, C_{α_i} and N_i.

$$V_\chi = \lambda_\chi \sum(\chi_i - \chi_0)^2 \tag{5}$$

$$\chi_i = \frac{(\boldsymbol{r}_{C'_i C_{\alpha_i}} \times \boldsymbol{r}_{C_{\alpha_i} N_i})}{|\boldsymbol{r}_{C'_i C_{\alpha_i}} \times \boldsymbol{r}_{C_{\alpha_i} N_i}|} \cdot \frac{\boldsymbol{r}_{C_{\alpha_i} C_{\beta_i}}}{|\boldsymbol{r}_{C_{\alpha_i} C_{\beta_i}}|} \tag{6}$$

1.4 Rama term

The rama term is used to fix the ϕ, ψ angles within a reasonable range.

$$V_{rama} = -\lambda_{rama}\sum_{i=2}^{N-1}\sum_{j} W_j e^{-\sigma_j(\omega_{\phi_j}(\cos(\phi_i - \phi^0_j) - 1)^2 + \omega_{\psi_j}(\cos(\psi_i - \psi^0_j) - 1)^2)} \tag{7}$$

The chirality term V_χ and Rama term was implemented using "CustomCompoundBondForce".

Table 1: parameters

Parameter	Value	Units
λ_{con}	120	kcal/$\AA^2$ mol
λ_{chain}	120	kcal/$\AA^2$ mol
λ_{χ}	60	kcal/ mol
λ_{rama}	2	kcal/ mol
λ_{excl}	20	kcal/$\AA^2$ mol
$r^0_{C\alpha_i C\alpha_{i+1}}$	3.816	$\AA$
$r^0_{C\alpha_i CO_i}$	2.40	$\AA$
$r^0_{CO_i C\alpha_i}$	2.76	$\AA$
$r^0_{C\alpha_i C\beta_i}$	1.53	$\AA$
$r^0_{N_i C\beta_i}$	2.46	$\AA$
$r^0_{C'_i C\beta_i}$	2.52	$\AA$
$r^0_{N_i C'_i}$	2.46	$\AA$
χ_0	-0.71	$\AA^3$

	General Case	Alpha Helix	Beta Sheet	Proline
W	1.3149 1.32016 1.0264	2.0	2.0	2.17 2.15
σ	15.398 49.0521 49.0954	419.0	15.398	105.52 109.09
ω_ϕ	0.15 0.25 0.65	1.0	1.0	1.0 1.0
ϕ_0	-1.74 -1.265 1.041	-0.895	-2.25	-1.153 -0.95
ω_ψ	0.65 0.45 0.25	1.0	1.0	0.15 0.15
ψ_0	2.138 -0.318 0.78	-0.82	2.16	2.4 -0.218

1.5 Excluded Volume term

The excluded volume term prevents the overlapping of backbone atoms.

$$V_{excl} = \lambda_{excl} \sum_{ij} [H(r_{C_iC_j} - r^C_{ex})(r_{C_iC_j} - r^C_{ex})^2 + H(r_{O_iO_j} - r^O_{ex})(r_{O_iO_j} - r^O_{ex})^2] \tag{8}$$

$$H(r) = \{ \begin{array}{cc} 1 & x \geq 0 \\ 0 & x \leq 0 \end{array} \tag{9}$$

The excluded volume term used "CustomNonbondedForce". All the parameters are the same as those defined in the original AWSEM paper. The parameters are defined in Table 1

1.6 Contact term

The transferable interactions have the form:

$$V_{contact} = V_{direct} + V_{water} \tag{10}$$

$$V_{direct} = \sum_{j-i>9} \gamma_{ij}(a_i, a_j)\Theta^I_{i,j} \tag{11}$$

$$V_{water}(i,j) = \sum_{j-i>9} \Theta^{II}_{i,j}(\sigma^{wat}_{ij}\gamma^{wat}_{ij}(a_i, a_j) + \sigma^{prot}_{ij}\gamma^{prot_{wat}}_{ij}(a_i, a_j)) \tag{12}$$

$$\Theta^{\mu}_{i,j} = \frac{1}{4}(1 + \tanh(\eta(r_{ij} - r^{\mu}_{min})))(1 + \tanh(\eta(r^{\mu}_{max} - r_{ij}))) \tag{13}$$

$$\sigma^{water}_{ij} = \frac{1}{4}(1 - \tanh(\eta_\sigma(\rho_i - \rho_0)))(1 - \tanh(\eta_\sigma(\rho_j - \rho_0))) \tag{14}$$

$$\sigma^{prot}_{ij} = 1 - \sigma^{water}_{ij} \tag{15}$$

1.7 β-hydrogen bonding and P-AP terms

We made some modification of these terms in order to make more efficient implementation of the force fields.

$$\theta_{i,j} = exp(-\frac{(r_{O_iN_j} - r_{ON})^2}{2\sigma^2_{ON}} - \frac{(r_{O_iH_j} - r_{OH})^2}{2\sigma^2_{OH}}) \tag{16}$$

$$\theta_{j,i} = exp(-\frac{(r_{O_jN_i} - r_{ON})^2}{2\sigma^2_{ON}} - \frac{(r_{O_jH_i} - r_{OH})^2}{2\sigma^2_{OH}}) \tag{17}$$

$$\theta_{j,i+2} = exp(-\frac{(r_{O_jN_{i+2}} - r_{ON})^2}{2\sigma^2_{ON}} - \frac{(r_{O_jH_{i+2}} - r_{OH})^2}{2\sigma^2_{OH}}) \tag{18}$$

$$V1_{ij} = \lambda_1(i,j)\theta_{i,j} \tag{19}$$

$$V2_{ij} = \lambda_2(i,j)\theta_{i,j}\theta_{j,i} \tag{20}$$

$$V3_{ij} = \lambda_3(i,j)\theta_{i,j}\theta_{j,i+2} \tag{21}$$

$$V_{ij} = V1_{ij} + V2_{ij} + V3_{ij} \tag{22}$$

$$V_{beta} = -k_{beta}\sum_{ij} V_{ij} \tag{23}$$

In previous the LAMMPS implementation, $V_{beta} = -k_{beta}\sum_{ij} V_{ij}v_iv_j$, the additional term v_iv_j was used to ensure that the hydrogen bonds do not occur within a span of 5 residues that is shorter than $12\AA$. Now this constraint is incorporated onto the pap term. The V_{beta} defined here can be fit into the "CustomHbondForce" template. Since for $V2_{ij}$, we can define O_i, N_i, H_i, the oxygen, hydrogen and nitrogen of residue i as the donor, and N_j, H_j, O_j as the acceptor. We could have implemented the exact same version as the LAMMPS version using "CustomCompoundBondForce", but computing bonded forces is much slower than computing non-bonded forces like "CustomHbondForce". When two residues are far apart, computing their interaction is unnecessary.

$$v_i = \frac{1}{2}(1 + \tanh(\mu_1 * (r_{ca_i ca_{i+4}} - rc_{HB}))) \quad (24)$$

$$\theta^1_{i,j} = \frac{1}{2}(1 + \tanh(\eta_{pap} * (r_0 - r_{ca_i n_j}))) \quad (25)$$

$$\theta^2_{i,j} = \frac{1}{2}(1 + \tanh(\eta_{pap} * (r_0 - r_{ca_{i+4} n_{j+4}}))) \quad (26)$$

$$\theta^3_{i,j} = \frac{1}{2}(1 + \tanh(\eta_{pap} * (r_0 - r_{ca_{i+4} n_{j-4}}))) \quad (27)$$

$$V_{i,j} = (\gamma_1(i,j) + \gamma_2(i,j)\theta^1_{i,j}\theta^2_{i,j} + \gamma_3 i, j\theta^3_{i,j})v_i \quad (28)$$

$$V_{pap} = \sum_{i,j} k_{pap} V_{i,j} \quad (29)$$

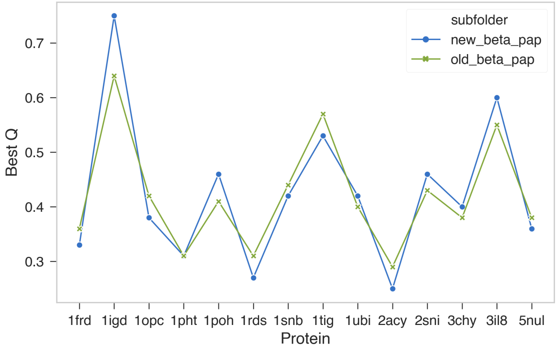

Figure 1: No significant different between structure prediction results using new and old beta hydrogen bonding term and pap term implementation.

2 Open3SPN2

The open3SPN2 software framework implements the 3SPN.2 [1] forcefields for A-DNA and B-DNA, and the 3SPN.2C [2] forcefield. The 3SPN.2 forcefield has been previously parametrized taking into account the free energy of nucleic acid hybridization, the intra strand base stacking energy, the DNA persistence length and the width of minor and major groves [1]. The 3SPN.2C forcefield is an extension of the 3SPN.2 forcefield that is able to reproduce sequence dependent curvature in the DNA [2].

In 3SPN.2 and 3SPN.2C each residue is represented by a three sites: a phosphate site (P), a sugar site (S) and a nucleobase site (B), where the nucleobase can be adenine (A), guanine (G), cytosine(C), or thymine (T). The 3SPN.2 potentials are the sum of eight terms (see equation 30). Three of this terms are bonded terms, which include a two-site bond term (V_{Bond}), a three-site angle term (V_{Angle}), and a four-site dihedral term ($V_{Dihedral}$). Another three terms depend on the angles between nucleobases. Among them is a three-site stacking term ($V_{Stacking}$) between consecutive nucleotides, a four-site basepairing term ($V_{BasePair}$) between complementary nucleobases, and a five-site cross-stacking

term ($V_{CrossStacking}$). The last two non-bonded terms depend only on the pairwise distances between sites and include an exclusion term ($V_{Exclusion}$) and an electrostatics term ($V_{Electrostatics}$).

$$\begin{aligned} V_{3SPN2} =& V_{Bond} + V_{Angle} + V_{Dihedral} + V_{Stacking} + V_{BasePair} \\ &+ V_{CrossStacking} + V_{Exclusion} + V_{Electrostatics} \end{aligned} \tag{30}$$

For 3SPN.2C a reference atomistic structure needs to be created using the 3DNA software [3]. The reference structure is given by a set of base-step and base-pair geometric parameters (Tables 2 and 3) suited for protein-DNA binding [2]. The base step-parameters depend on the type of the base (B_o) and the type of the neighboring sequence-adjacent base (B_n). The base-pair parameters depend only on the type of the base (B_o), since we expect a Watson-Crick basepair (B_p) After the atomistic reference structure is created, the structure is Coarse Grained and the distances, angles and dihedrals from the structure will become the equilibrium distances, angles and dihedrals for the 3SPN.2C forcefield.

Table 2: open3SPN2 base-step reference geometric parameters

B_o	B_n	**twist** (°)	**roll** (°)	**tilt** (°)	**shift** ($\AA$)	**slide** ($\AA$)	**rise** ($\AA$)
A	A	35.31	0.76	−1.84	−0.05	−0.21	3.27
A	T	31.21	−1.39	0	0	−0.56	3.39
A	C	31.52	0.91	−0.64	0.21	−0.54	3.39
A	G	33.05	3.15	−1.48	0.12	−0.27	3.38
T	A	36.2	5.25	0	0	0.03	3.34
T	T	35.31	0.76	1.84	0.05	−0.21	3.27
T	C	34.8	3.87	1.52	0.27	−0.03	3.35
T	G	35.02	5.95	0.05	0.16	0.18	3.38
C	A	35.02	5.95	−0.05	−0.16	0.18	3.38
C	T	33.05	3.15	1.48	−0.12	−0.27	3.38
C	C	33.17	3.86	0.4	0.02	−0.47	3.28
C	G	35.30	4.29	0	0	0.57	3.49
G	A	34.8	3.87	−1.52	−0.27	−0.03	3.35
G	T	31.52	0.91	0.64	−0.21	−0.54	3.39
G	C	34.38	0.67	0	0	−0.07	3.38
G	G	33.17	3.86	−0.4	−0.02	−0.47	3.28

Table 3: open3SPN2 base-pair reference geometric parameters

B_o	B_p	**buckle** (°)	**propeller** (°)	**opening** (°)	**shear** ($\AA$)	**stretch** ($\AA$)	**stagger** ($\AA$)
A	T	1.8	−15	1.5	0.07	−0.19	0.07
T	A	−1.8	−15	1.5	−0.07	−0.19	0.07
C	G	−4.9	−8.7	−0.6	0.16	−0.17	0.15
G	C	4.9	−8.7	−0.6	−0.16	−0.17	0.15

2.1 Bonded terms

The bond term is a quartic function of the pairwise distance between two sites that doesn't include the cubic term. The coefficient for the quartic term is 100 times greater per $\mathring{A}^2$ than the coefficient for the harmonic term (Eq. 31). The quartic function allows the bond to have a wider well than an harmonic potential with a comparable coefficient (Figure 2).

$$V_{Bond} = \sum_{i}^{Bonds} k_{b_i}(r_{bi} - r_{bi}^{o})^2 + 100k_{b_i}(r_{bi} - r_{bi}^{o})^4 \tag{31}$$

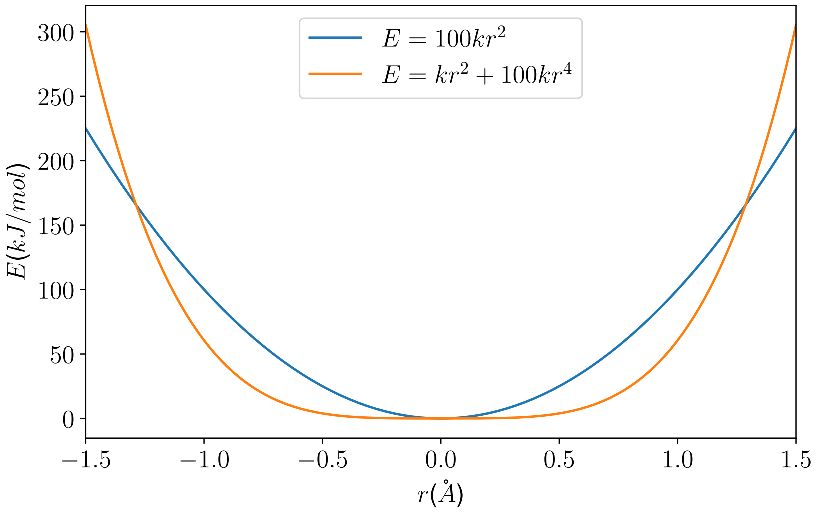

Figure 2: The harmonic potential function is shown in blue, compared with the open3SPN2 quartic potential function with $k_b = 0.143403 kcal/mol/\mathring{A}$. The open3SPN2 bond potential function shows a wider well than a comparable harmonic potential function.

There are 6 types of bonds defined for each forcefield: a bond from a phosphate (P) to a sugar(S), a bond between a sugar (S) and the phosphate of the next residue (P_1), and four bonds from the sugar to the nucleobase that depend on the nucleobase type (Figure 3).

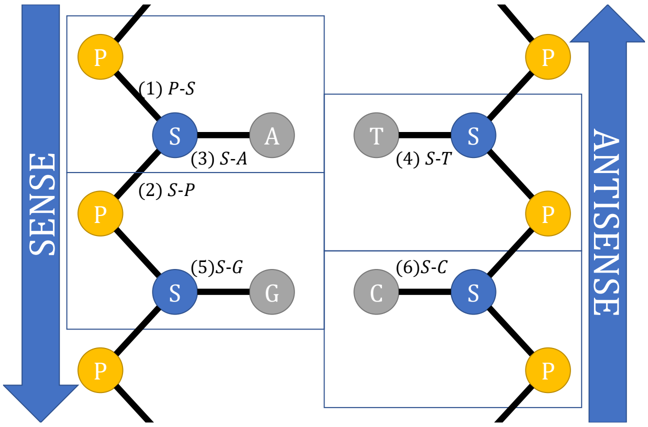

Figure 3: List of bonds in the 3SPN.2 and 3SPN.2C forcefields. Each square encloses a residue, which contains a phosphate site (P), a sugar site (S) and a nucleobase site (A, C, T, or G). The 6 types or bonds are listed from 1 to 6.

The parameters of the bonds are listed on the table 4, where i is the first site type, j is the second site type, r_b^o is the equilibrium distance of the bond, and k_b is the coefficient for the harmonic term. In 3SPN.2C the equilibrium distances (r_b^o) are computed from a reference structure generated using the equilibrium base-pair and base-step parameters, so they are not shown in the table.

Table 4: open3SPN2 bond parameters

**Forcefield**	i	j	$r_b^o(\AA)$	$k_b(kcal/mol/\AA^2)$
3SPN.2 (A-DNA)	P	S	4.157	0.143 403
3SPN.2 (A-DNA)	S	P_1	3.78	0.143 403
3SPN.2 (A-DNA)	S	A	4.697	0.143 403
3SPN.2 (A-DNA)	S	T	4.22	0.143 403
3SPN.2 (A-DNA)	S	G	4.852	0.143 403
3SPN.2 (A-DNA)	S	C	4.066	0.143 403
3SPN.2 (B-DNA)	P	S	3.899	0.143 403
3SPN.2 (B-DNA)	S	P_1	3.559	0.143 403
3SPN.2 (B-DNA)	S	A	4.67	0.143 403
3SPN.2 (B-DNA)	S	T	4.189	0.143 403
3SPN.2 (B-DNA)	S	G	4.829	0.143 403
3SPN.2 (B-DNA)	S	C	4.112	0.143 403
3SPN.2C	P	S	—	0.143 403
3SPN.2C	S	P_1	—	0.143 403
3SPN.2C	S	A	—	0.143 403
3SPN.2C	S	T	—	0.143 403
3SPN.2C	S	G	—	0.143 403
3SPN.2C	S	C	—	0.143 403

1 The suffix 1 in the names of the sites indicates that the site is part of the next residue.
— The equilibrium distances (r_b^o) for the 3SPN.2C forcefield is sequence dependent and computed from a template created based on the geometric parameters.

The angle term is an harmonic function of the angle θ_a between 3 sites i, j and k, where j is the center site. The term coefficient (k_a) is 200 $kJ/mol/rad^2$ in the 3SPN.2 forcefield. There are 10 possible angles: P-S-P, S-P-S, P-S-B and B-P-S, where B can be any nucleobase (A,C,T,G) (Figure 4 and their parameters are listed in the table 5.

$$V_{Angle} = \sum_{i}^{Angles} k_{ai}(\theta_{ai} - \theta_{ai}^{o})^2 \qquad (32)$$

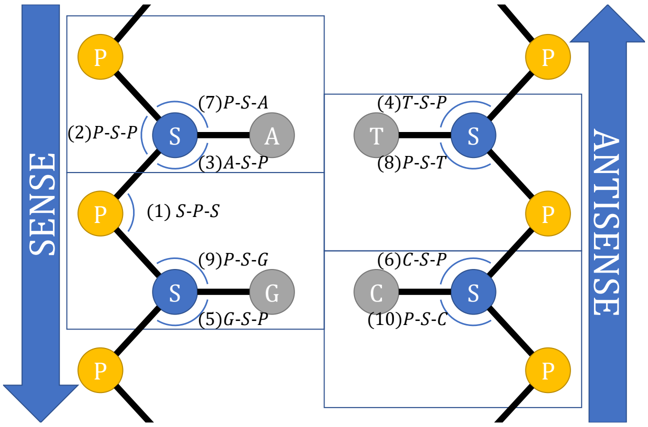

Figure 4: List of angles in the 3SPN.2 and 3SPN.2C forcefields. Each square encloses a residue, which contains a phosphate site (P), a sugar site (S) and a nucleobase site (A, C, T, or G). The 10 types of angles are listed from 1 to 10.

Table 5: Open3SPN.2 angle parameters

**Forcefield**	**i**	**j**	**k**	θ_a^o (°)
3SPN.2 (A-DNA)	S	P_1	S_1	92.77
3SPN.2 (A-DNA)	P	S	P_1	91.24
3SPN.2 (A-DNA)	A	S	P_1	104.86
3SPN.2 (A-DNA)	T	S	P_1	110.58
3SPN.2 (A-DNA)	G	S	P_1	103.86
3SPN.2 (A-DNA)	C	S	P_1	106.94
3SPN.2 (A-DNA)	P	S	A	103.71
3SPN.2 (A-DNA)	P	S	T	93.27
3SPN.2 (A-DNA)	P	S	G	107.49
3SPN.2 (A-DNA)	P	S	C	97.58
3SPN.2 (B-DNA)	S	P_1	S_1	94.49
3SPN.2 (B-DNA)	P	S	P_1	120.15
3SPN.2 (B-DNA)	A	S	P_1	112.07
3SPN.2 (B-DNA)	T	S	P_1	116.68
3SPN.2 (B-DNA)	G	S	P_1	110.12
3SPN.2 (B-DNA)	C	S	P_1	114.34
3SPN.2 (B-DNA)	P	S	A	103.53
3SPN.2 (B-DNA)	P	S	T	92.06
3SPN.2 (B-DNA)	P	S	G	107.4
3SPN.2 (B-DNA)	P	S	C	96.96

In 3SPN.2C the equilibrium angles (θ_a^o) are computed from a reference structure generated using the equilibrium base-pair and base-step parameters. The equilibrium constant also depends on the nucleobase type (B_o) and the neighboring bases (B_n) as shown in the table 7.

Table 7: Open3SPN.2C angle parameters

**Forcefield**	**i**	**j**	**k**	$k_a kcal/mol/rad^2$	θ_a^o (°)	B_o	B_n
3SPN.2C	S	P_1	S_1	355	—	A	A
3SPN.2C	S	P_1	S_1	464	—	A	C
3SPN.2C	S	P_1	S_1	368	—	A	G
3SPN.2C	S	P_1	S_1	147	—	A	T
3SPN.2C	S	P_1	S_1	273	—	C	A
3SPN.2C	S	P_1	S_1	165	—	C	C
3SPN.2C	S	P_1	S_1	478	—	C	G
3SPN.2C	S	P_1	S_1	368	—	C	T
3SPN.2C	S	P_1	S_1	442	—	G	A
3SPN.2C	S	P_1	S_1	228	—	G	C
3SPN.2C	S	P_1	S_1	165	—	G	G
3SPN.2C	S	P_1	S_1	464	—	G	T
3SPN.2C	S	P_1	S_1	230	—	T	A
3SPN.2C	S	P_1	S_1	442	—	T	C
3SPN.2C	S	P_1	S_1	273	—	T	G
3SPN.2C	S	P_1	S_1	355	—	T	T
3SPN.2C	P	S	P_1	300	—	any	any
3SPN.2C	A	S	P_1	460	—	A	A
3SPN.2C	A	S	P_1	442	—	A	C
3SPN.2C	A	S	P_1	358	—	A	G
3SPN.2C	A	S	P_1	370	—	A	T
3SPN.2C	T	S	P_1	120	—	T	A
3SPN.2C	T	S	P_1	383	—	T	C
3SPN.2C	T	S	P_1	206	—	T	G
3SPN.2C	T	S	P_1	460	—	T	T
3SPN.2C	G	S	P_1	383	—	G	A
3SPN.2C	G	S	P_1	336	—	G	C
3SPN.2C	G	S	P_1	278	—	G	G
3SPN.2C	G	S	P_1	442	—	G	T
3SPN.2C	C	S	P_1	206	—	C	A
3SPN.2C	C	S	P_1	278	—	C	C
3SPN.2C	C	S	P_1	278	—	C	G
3SPN.2C	C	S	P_1	358	—	C	T
3SPN.2C	P	S	A	460	—	A	A_{-1}
3SPN.2C	P	S	A	206	—	A	C_{-1}
3SPN.2C	P	S	A	383	—	A	G_{-1}
3SPN.2C	P	S	A	120	—	A	T_{-1}
3SPN.2C	P	S	T	370	—	T	A_{-1}
3SPN.2C	P	S	T	358	—	T	C_{-1}
3SPN.2C	P	S	T	442	—	T	G_{-1}
3SPN.2C	P	S	T	460	—	T	T_{-1}
3SPN.2C	P	S	G	358	—	G	A_{-1}
3SPN.2C	P	S	G	278	—	G	C_{-1}
3SPN.2C	P	S	G	278	—	G	G_{-1}
3SPN.2C	P	S	G	206	—	G	T_{-1}
3SPN.2C	P	S	C	442	—	C	A_{-1}

Table 7: Open3SPN.2C angle parameters (continued)

**Forcefield**	**i**	**j**	**k**	$k_a(kJ/mol)$	θ_a^o (°)	B_o	B_n
3SPN.2C	P	S	C	278	—	C	C_{-1}
3SPN.2C	P	S	C	336	—	C	G_{-1}
3SPN.2C	P	S	C	383	—	C	T_{-1}

1 The suffix 1 in the names of the sites indicates that the site is part of the next residue.
−1 The neighboring base on the 5' direction or behind in the sequence.
— The equilibrium angles (θ_a^o) for the 3SPN.2C forcefield is sequence dependent and computed from a template based on the geometric parameters.

The open3SPN2 forcefield includes two dihedral potentials, a gaussian potential and a cosine potential (Eq. 33).

$$V_{Dihedral} = \sum_{i}^{Dihedrals} -k_{Gi} e^{\frac{-(\phi_i - \phi_i^o)^2}{2\sigma_i^2}} + k_{Ci}(1 - cos(\phi_i - \phi_i^o)) \tag{33}$$

Where k_G is the coefficient for the gaussian potential, k_C is the coefficient for the cosine potential, ϕ is the dihedral angle between the sites i, j, k, and l. The parameters are listed in the table 8.

In 3SPN.2 only the gaussian potential is used, while in 3SPN.2C a mixture of the gaussian potential and the cosine potential is used for the dihedrals S-P-S-P and P-S-P-S. 3SPN.2C also adds a dihedral potential for the dihedrals B-S-P-S and S-P-S-B, where B can be any nucleobase (Figure 5). In 3SPN.2C the equilibrium angles (ϕ_D^o) are computed from a template structure generated using the equilibrium basepair and base stacking parameters.

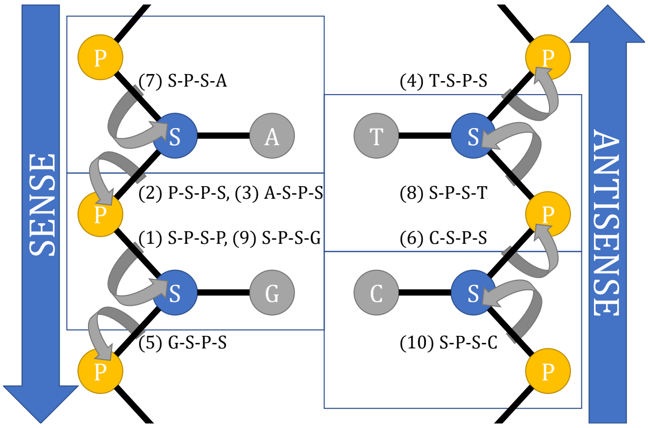

Figure 5: List of dihedrals in the 3SPN.2 and 3SPN.2C forcefields. Each square encloses a residue, which contains a phosphate site (P), a sugar site (S) and a nucleobase site (A, C, T, or G). The 10 types or dihedrals are listed from 1 to 10. 3SPN.2 only includes the dihedrals (1) and (2).

Table 8: Open3SPN.2C dihedral parameters

**Forcefield**	**i**	**j**	**k**	**l**	$K_C(kcal/mol)$	$K_G(kcal/mol)$	$\sigma(rad)$	ϕ^o (°)
3SPN.2 (A-DNA)	S	P_1	S_1	P_2	0	1.434034	0.3	-9.58
3SPN.2 (A-DNA)	P	S	P_1	S_1	0	1.434034	0.3	-328.4
3SPN.2 (B-DNA)	S	P_1	S_1	P_2	0	1.434034	0.3	-359.17
3SPN.2 (B-DNA)	P	S	P_1	S_1	0	1.434034	0.3	-334.79
3SPN.2C	S	P_1	S_1	P_2	0.478011	1.67304	0.3	—
3SPN.2C	P	S	P_1	S_1	0.478011	1.67304	0.3	—
3SPN.2C	A	S	P_1	S_1	0.478011	0	0.3	—
3SPN.2C	T	S	P_1	S_1	0.478011	0	0.3	—
3SPN.2C	G	S	P_1	S_1	0.478011	0	0.3	—
3SPN.2C	C	S	P_1	S_1	0.478011	0	0.3	—
3SPN.2C	S	P_1	S_1	A_1	0.478011	0	0.3	—
3SPN.2C	S	P_1	S_1	T_1	0.478011	0	0.3	—
3SPN.2C	S	P_1	S_1	G_1	0.478011	0	0.3	—
3SPN.2C	S	P_1	S_1	C_1	0.478011	0	0.3	—

1 The suffix 1 in the names of the sites indicates that the site is part of the next residue.
— The equilibrium dihedral angles (ϕ^o) for the 3SPN.2C forcefield is sequence dependent and computed from a template based on the geometric parameters.

2.2 Stacking, BasePairing and CrossStacking terms

The Stacking, BasePairing and CrossStacking terms are non-bonded tems that depend on the distance between the nucleobases (r_{BS}, r_{BP}, and r_{CS} respectively), as well as angles defined between the residues. All the terms include a modulating function (f) of an angle (θ). The modulating function can be understood as depending in the position of the second nucleobase relative to two cones in 3D space. If the second nucleobase is inside the interior cone, the modulating function is equal to 1, and if it is outside the cone, the modulating function is equal to 0. Between this two cones the modulating function has a value between 1 and 0 that depends on the angle (Eq 34). The coefficient K defines the width of the conical section.

$$f(\theta|K,\theta^o) = \begin{cases} 1 & \frac{\pi}{2} \geqslant |K(\theta-\theta^o)| \\ 1 - cos^2(K(\theta-\theta^o)) & \frac{\pi}{2} < |K(\theta-\theta^o)| \leqslant \pi \\ 0 & \pi < |K(\theta-\theta^o)| \end{cases} \tag{34}$$

For the stacking term $K_{BS} = 6$, which defines a inner cone of 30 degrees and an outer cone of 60 degrees. The stacking potential is a mixture of a repulsive potential and an attractive potential. The depth of the attractive well is ϵ and fluctuates with the modulating function. The steepness of the repulsive potential is $\alpha_{BS} = 3\mathring{A}^{-2}$. The parameters are listed in the table 9.

$$F_{BS}(\theta_{BS}) = f(\theta_{BS}|K_{BS},\theta^o_{BS}) \tag{35}$$

$$V_{BS} = \sum_i \begin{cases} \epsilon_i (1 - e^{-\alpha_{BS}(r_{BS\,i} - r_{BS}{}_i^o)^2}) - \epsilon_i F_{BS}(\theta_{BS\,i}) & , r_{BS\,i} < r_{BS}{}_i^o \\ \epsilon_i (1 - e^{-\alpha_{BS}(r_{BS\,i} - r_{BS}{}_i^o)^2}) F_{BS}(\theta_{BS\,i}) - \epsilon_i F_{BS}(\theta_{BS\,i}) & , r_{BS\,i} \geqslant r_{BS}{}_i^o \end{cases} \tag{36}$$

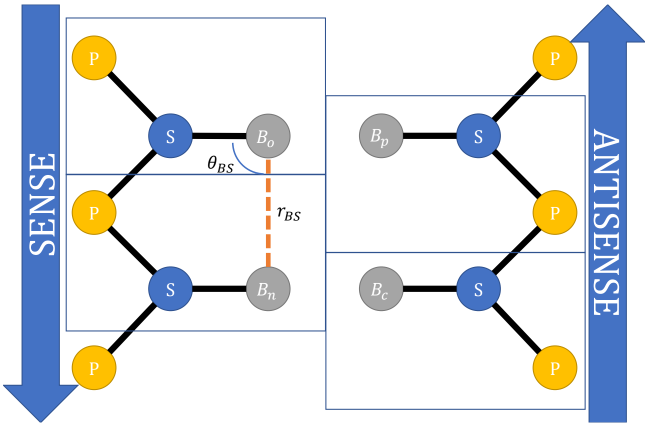

Figure 6: Important variables used for the stacking term. Each square encloses a residue, which contains a phosphate site (P), a sugar site (S) and a nucleobase site. The nucleobases shown are the reference nucleobase (B_o), the neighboring nucleobase (B_n), the base-pairing nucleobase (B_p) and the cross-stacking nucleobase (B_c). The variables shown are the distance between B_o and B_n (r_{BS}), and the angle between S, B_o, and B_n (θ_{BS}).

Table 9: open3SPN2 base stacking parameters

**DNA**	B_o	B_n	$\epsilon(kcal/mol)$	$r_B S^o(\AA)$	$\theta^o_{BS}(^\circ)$
3SPN.2 (A-DNA)	A	A	3.439293	4.022	108.32
3SPN.2 (A-DNA)	A	T	3.427342	3.344	96.74
3SPN.2 (A-DNA)	A	G	3.166826	4.261	111.32
3SPN.2 (A-DNA)	A	C	3.467973	3.737	97.36
3SPN.2 (A-DNA)	T	A	2.478489	4.794	103.33
3SPN.2 (A-DNA)	T	T	3.193117	4.031	94.85
3SPN.2 (A-DNA)	T	G	2.471319	5.064	105.36
3SPN.2 (A-DNA)	T	C	3.080784	4.445	94.51
3SPN.2 (A-DNA)	G	A	3.539675	3.855	108.25
3SPN.2 (A-DNA)	G	T	3.721319	3.217	95.59
3SPN.2 (A-DNA)	G	G	3.568356	4.077	111.66
3SPN.2 (A-DNA)	G	C	3.678298	3.592	96.71
3SPN.2 (A-DNA)	C	A	2.729446	4.499	111.39
3SPN.2 (A-DNA)	C	T	3.056883	3.708	102.73
3SPN.2 (A-DNA)	C	G	2.51434	4.772	113.47
3SPN.2 (A-DNA)	C	C	3.164436	4.116	102.14
3SPN.2 (B-DNA)	A	A	3.439293	3.716	101.15
3SPN.2 (B-DNA)	A	T	3.427342	3.675	85.94

3SPN.2 (B-DNA)	A	G	3.166826	3.827	105.26
3SPN.2 (B-DNA)	A	C	3.467973	3.744	89
3SPN.2 (B-DNA)	T	A	2.478489	4.238	101.59
3SPN.2 (B-DNA)	T	T	3.193117	3.984	89.5
3SPN.2 (B-DNA)	T	G	2.471319	4.416	104.31
3SPN.2 (B-DNA)	T	C	3.080784	4.141	91.28
3SPN.2 (B-DNA)	G	A	3.539675	3.576	100.89
3SPN.2 (B-DNA)	G	T	3.721319	3.598	84.83
3SPN.2 (B-DNA)	G	G	3.568356	3.664	105.48
3SPN.2 (B-DNA)	G	C	3.678298	3.635	88.28
3SPN.2 (B-DNA)	C	A	2.729446	4.038	106.49
3SPN.2 (B-DNA)	C	T	3.056883	3.798	93.31
3SPN.2 (B-DNA)	C	G	2.51434	4.208	109.54
3SPN.2 (B-DNA)	C	C	3.164436	3.935	95.46
3SPN.2C	A	A	3.303059	3.58	100.13
3SPN.2C	A	T	3.597036	3.56	90.48
3SPN.2C	A	G	3.183556	3.85	104.39
3SPN.2C	A	C	3.781071	3.45	93.23
3SPN.2C	T	A	2.186902	4.15	102.59
3SPN.2C	T	T	2.973231	3.93	93.32
3SPN.2C	T	G	2.289675	4.32	103.7
3SPN.2C	T	C	3.133365	3.87	94.55
3SPN.2C	G	A	3.288719	3.51	95.45
3SPN.2C	G	T	3.487094	3.47	87.63
3SPN.2C	G	G	3.530115	3.67	106.36
3SPN.2C	G	C	3.625717	3.42	83.12
3SPN.2C	C	A	2.210803	4.15	102.69
3SPN.2C	C	T	2.968451	3.99	96.05
3SPN.2C	C	G	2.110421	4.34	100.46
3SPN.2C	C	C	3.34847	3.84	100.68

The modulating function for the base pair term depends on the angles between the sugar (S), the base(B_o) and the complementary base(B_n). There are two angles that can be defined on this way(θ_{BP1},θ_{BP2}). It also depends on the cosine of the dihedral between both sugars and bases (S-B-B-S) (ϕ_{BP}). The cone for basepairing is much narrower ($K_{BP} = 12$) where the inner cone is 15 degrees and the outer cone is 30 degrees.

$$F_{BP} = \frac{1 + cos(\phi_{BP})}{2} f(\theta_{BP1}|K_{BP}, {\theta_{BP1}}_1^o) f(\theta_{BP1}|K_{BP}, {\theta_{BP1}}_2^o) \tag{37}$$

The steepness parameter, α_{BP}, is $2nm^{-2}$. The parameters are listed on the table 10.

$$V_{BP} = \sum_i \begin{cases} \epsilon_i(1 - e^{-\alpha_{BP}(r_{BPi} - r_{BP}{}_i^o)^2}) - \epsilon_i F_{BP} & , r_{BPi} < r_{BP}{}_i^o \\ \epsilon_i(1 - e^{-\alpha_{BP}(r_{BPi} - r_{BP}{}_i^o)^2}) F_{BP} - \epsilon_i F_{BP} & , r_{BPi} \geqslant r_{BP}{}_i^o \end{cases} \tag{38}$$

Table 10: open3SPN2 basepairing parameters

**Forcefield**	B_o	B_p	r^o_{BP} ($\AA$)	ϵ_{BP} ($kcal/mol$)	ϕ_{BP} (°)	θ_{BP1} (°)	θ_{BP2} (°)
3SPN.2 (A-DNA)	A	T	5.861	3.99874	50.17	160.91	140.49
3SPN.2 (A-DNA)	G	C	5.528	5.06241	38.33	165.25	147.11
3SPN.2 (B-DNA)	A	T	5.941	3.99874	-38.35	156.54	135.78
3SPN.2 (B-DNA)	G	C	5.530	5.06241	-42.98	159.81	141.16
3SPN.2C	A	T	5.82	3.44292	-38.18	153.17	133.51
3SPN.2C	G	C	5.52	4.35873	-35.75	159.5	138.08

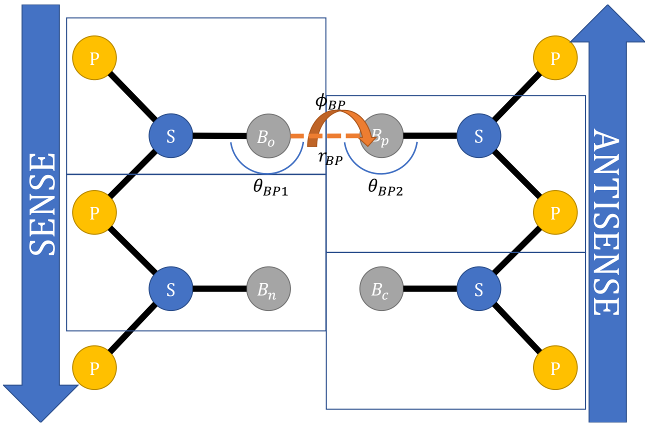

Figure 7: Important variables used for the base-pairing term. Each square encloses a residue, which contains a phosphate site (P), a sugar site (S) and a nucleobase site. The nucleobases shown are the reference nucleobase (B_o), the neighboring nucleobase (B_n), the base-pairing nucleobase (B_p) and the cross-stacking nucleobase (B_c). The variables shown are the distance between B_o and B_p (r_{BP}), the angle between the sugar from the the reference nucleotide, B_o, and B_p (θ_{BP1}), the angle between the sugar from the the base-pairing nucleotide, B_p, and B_o (θ_{BP2}), and the dihedral between the sugar from the the reference nucleotide, B_o, and B_p and the sugar from the the base-pairing nucleotide (ϕ_{BP}).

The CrossStacking potential has only an attractive potential. the modulating function depends on the angle between the sugar, the base, and the cross-stacking base. It also depends on the vector angle between defined between the sugar-base vectors. K for the first angle is 8, while for the second angle is 12. α is $2nm^{-2}$.

$$V_{CS} = \sum_i \begin{cases} -\epsilon_i F_{CS} & , r_{CS\,i} < r_{CS\,i}^o \\ \epsilon_i (1 - e^{-\alpha_i (r_{CS\,i} - r_{CS\,i}^o)^2}) F_{CS} - \epsilon_i F_{CS} & , r_{CS\,i} \geqslant r_{CS\,i}^o \end{cases} \tag{39}$$

$$F_{CS} = f(\phi_{CS} | K_{BP}, \phi_{CS}{}^o) f(\theta_{CS} | K_{CS}, \theta^o_{CS}) \tag{40}$$

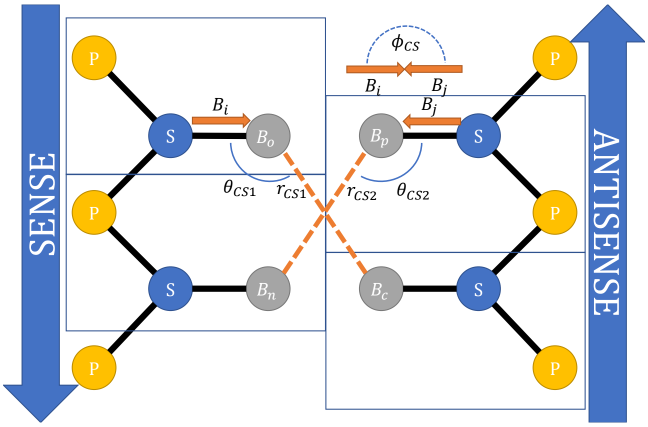

Figure 8: Important variables used for the cross-stacking term. Each square encloses a residue, which contains a phosphate site (P), a sugar site (S) and a nucleobase site. The nucleobases shown are the reference nucleobase (B_o), the neighboring nucleobase (B_n), the base-pairing nucleobase (B_p) and the cross-stacking nucleobase (B_c). Some variables shown are the distance between B_o and B_c (r_{CS1}), the angle between S, B_o, and B_c (θ_{CS1}). This variables are also mirrored for the base-pairing nucleotide. Also shown are the vectors B_i and B_j, which are defined as the vectors originating from the sugar to the nucleobase of the reference nucleotide and the base-pairing nucleotide respectively. ϕ_{CS} is the vector angle between B_i and B_j.

Table 11: open3SPN2 CrossStacking parameters

**Forcefield**	B_o	B_p	B_c	ϕ^o_{CS} (°)	θ_{CS1} (°)	θ_{CS2} (°)	r_{CS1} (Å)	r_{CS2} (Å)	ϵ_{CS1} ($kcal/mol$)	ϵ_{CS2} ($kcal/mol$)
3SPN.2 (A-DNA)	A	T	A	126.57	147.44	130.5	7.344	4.624	0.522452	0.522452
3SPN.2 (A-DNA)	A	T	T	126.57	148.97	138.73	8.081	5.095	0.662942	0.662942
3SPN.2 (A-DNA)	A	T	G	126.57	146.21	126.68	7.187	4.464	0.677075	0.712197
3SPN.2 (A-DNA)	A	T	C	126.57	150.17	134.18	7.99	5.162	0.46634	0.60683
3SPN.2 (A-DNA)	T	A	A	126.57	138.42	130.41	8.081	5.095	0.662942	0.662942
3SPN.2 (A-DNA)	T	A	T	126.57	141.67	134.68	8.755	5.693	0.522452	0.522452
3SPN.2 (A-DNA)	T	A	G	126.57	136.64	127.69	7.952	4.896	0.60683	0.46634
3SPN.2 (A-DNA)	T	A	C	126.57	141.64	131.38	8.697	5.724	0.712197	0.677075
3SPN.2 (A-DNA)	G	C	A	134.71	147.67	130.57	7.187	4.464	0.677075	0.712197
3SPN.2 (A-DNA)	G	C	T	134.71	148.28	140.17	7.952	4.896	0.60683	0.46634
3SPN.2 (A-DNA)	G	C	G	134.71	146.84	126.44	7.019	4.315	0.901943	1.1478
3SPN.2 (A-DNA)	G	C	C	134.71	150.02	135.31	7.844	4.968	0.269738	0.269738
3SPN.2 (A-DNA)	C	G	A	134.71	145.83	132.69	7.99	5.162	0.46634	0.60683
3SPN.2 (A-DNA)	C	G	T	134.71	148.39	138.21	8.697	5.724	0.712197	0.677075
3SPN.2 (A-DNA)	C	G	G	134.71	144.24	129.73	7.844	4.968	0.269738	0.269738
3SPN.2 (A-DNA)	C	G	C	134.71	148.74	134.45	8.63	5.759	1.1478	0.901943
3SPN.2 (B-DNA)	A	T	A	116.09	154.38	116.88	6.208	5.435	0.522452	0.522452
3SPN.2 (B-DNA)	A	T	T	116.09	159.1	121.74	6.876	6.295	0.662942	0.662942
3SPN.2 (B-DNA)	A	T	G	116.09	152.46	114.23	6.072	5.183	0.677075	0.712197
3SPN.2 (B-DNA)	A	T	C	116.09	158.38	119.06	6.811	6.082	0.46634	0.60683
3SPN.2 (B-DNA)	T	A	A	116.09	147.1	109.42	6.876	6.295	0.662942	0.662942
3SPN.2 (B-DNA)	T	A	T	116.09	153.79	112.95	7.48	7.195	0.522452	0.522452
3SPN.2 (B-DNA)	T	A	G	116.09	144.44	107.32	6.771	6.028	0.60683	0.46634
3SPN.2 (B-DNA)	T	A	C	116.09	151.48	110.56	7.453	6.981	0.712197	0.677075
3SPN.2 (B-DNA)	G	C	A	124.93	154.69	119.34	6.072	5.183	0.677075	0.712197
3SPN.2 (B-DNA)	G	C	T	124.93	157.83	124.72	6.771	6.028	0.60683	0.46634
3SPN.2 (B-DNA)	G	C	G	124.93	153.43	116.51	5.921	4.934	0.901943	1.1478
3SPN.2 (B-DNA)	G	C	C	124.93	158.04	121.98	6.688	5.811	0.269738	0.269738
3SPN.2 (B-DNA)	C	G	A	124.93	152.99	114.6	6.811	6.082	0.46634	0.60683
3SPN.2 (B-DNA)	C	G	T	124.93	159.08	118.26	7.453	6.981	0.712197	0.677075
3SPN.2 (B-DNA)	C	G	G	124.93	150.53	112.45	6.688	5.811	0.269738	0.269738
3SPN.2 (B-DNA)	C	G	C	124.93	157.17	115.88	7.409	6.757	1.1478	0.901943
3SPN.2C	A	T	A	110.92	154.04	116.34	6.42	5.58	0.449831	0.449831
3SPN.2C	A	T	T	110.92	158.77	119.61	6.77	6.14	0.570793	0.570793
3SPN.2C	A	T	G	110.92	153.88	115.19	6.27	5.63	0.582961	0.613202
3SPN.2C	A	T	C	110.92	157.69	120.92	6.84	6.18	0.401518	0.52248
3SPN.2C	T	A	A	110.92	148.62	107.4	6.77	6.14	0.570793	0.570793
3SPN.2C	T	A	T	110.92	155.05	110.76	7.21	6.8	0.449831	0.449831
3SPN.2C	T	A	G	110.92	147.54	106.33	6.53	6.07	0.52248	0.401518
3SPN.2C	T	A	C	110.92	153.61	111.57	7.08	6.64	0.613202	0.582961
3SPN.2C	G	C	A	120.45	153.91	121.61	6.27	5.63	0.582961	0.613202
3SPN.2C	G	C	T	120.45	155.72	124.92	6.53	6.07	0.52248	0.401518
3SPN.2C	G	C	G	120.45	151.84	120.52	5.74	5.87	0.776573	0.988256
3SPN.2C	G	C	C	120.45	157.8	124.88	6.86	5.66	0.232244	0.232244
3SPN.2C	C	G	A	120.45	152.04	112.45	6.84	6.18	0.401518	0.52248
3SPN.2C	C	G	T	120.45	157.72	115.43	7.08	6.64	0.613202	0.582961
3SPN.2C	C	G	G	120.45	151.65	110.51	6.86	5.66	0.232244	0.232244
3SPN.2C	C	G	C	120.45	154.49	115.8	6.79	6.8	0.988256	0.776573

2.3 Non-bonded terms

The exclussion potential contains the repulsive section of a lennard jones potential.

$$V_{Exclusion} = \sum_{ij} \begin{cases} \epsilon_r \left[\left(\frac{\sigma_{ij}}{r_{ij}} \right)^{12} - 2 \left(\frac{\sigma_{ij}}{r_{ij}} \right)^{6} \right] + \epsilon_r & , r < \sigma_{ij} \\ 0 & , r \geqslant \sigma_{ij} \end{cases} \tag{41}$$

The electrostatics potential is based on the Debye-Huckel potential.

$$V_{Electrostatics} = \sum_{ij} \frac{q_i q_j e^{-\frac{r_{ij}}{\lambda_D}}}{4\pi\epsilon_0 \epsilon \left(T, C\right) r_{ij}} \tag{42}$$

The dielectric coefficient depends on the temperature and the concentration of ions.

$$\lambda_D = \frac{\sqrt{\epsilon_0 \epsilon \left(T, C\right) r_{i,j}}}{2\beta N_A e_c^2 I} \tag{43}$$

Table 12: open3SPN2 Exclussion and electrostatics parameters

**Forcefield**	Particle	ϵ $(kcal/mol)$	r $(\AA)$	mass (Da)	charge (e)
3SPN.2 (A-DNA)	P	0.239006	4.5	94.9696	-0.6
3SPN.2 (A-DNA)	S	0.239006	6.2	83.1104	0
3SPN.2 (A-DNA)	A	0.239006	4.46	134.122	0
3SPN.2 (A-DNA)	T	0.239006	5.5	125.1078	0
3SPN.2 (A-DNA)	G	0.239006	4.2	150.1214	0
3SPN.2 (A-DNA)	C	0.239006	5.7	110.0964	0
3SPN.2 (B-DNA)	P	0.239006	4.5	94.9696	-0.6
3SPN.2 (B-DNA)	S	0.239006	6.2	83.1104	0
3SPN.2 (B-DNA)	A	0.239006	5.4	134.122	0
3SPN.2 (B-DNA)	T	0.239006	7.1	125.1078	0
3SPN.2 (B-DNA)	G	0.239006	4.9	150.1214	0
3SPN.2 (B-DNA)	C	0.239006	6.4	110.0964	0
3SPN.2C	P	0.239006	4.5	94.9696	-0.6
3SPN.2C	S	0.239006	6.2	83.1104	0
3SPN.2C	A	0.239006	5.4	134.122	0
3SPN.2C	T	0.239006	7.1	125.1078	0
3SPN.2C	G	0.239006	4.9	150.1214	0
3SPN.2C	C	0.239006	6.4	110.0964	0

2.4 Protein-DNA Excluded Volume term

To prevent protein and DNA overlap each other, we added a Lennard-Jones interaction between protein atoms and protein atoms.

$$V_{LJ}(r) = \begin{cases} 4\epsilon[(\frac{\sigma}{r})^{12} - (\frac{\sigma}{r})^{6}] - E_{cut} & r < r_c \\ 0 & r \geq r_c \end{cases} \tag{44}$$

with $\epsilon = 0.03\ kcal/mol$, $\sigma = 5.7\AA$, $r_c = 2.5\sigma$ and $E_{cut} = 4\epsilon[(\sigma/r_c)^{12} - (\sigma/r_c)^{6}]$ The detail of calibration of parameters for this term and the next term can be found in the SI of ref [4].

2.5 Protein-DNA Electrostatics term

The protein and DNA electrostatic interaction is modeled as a Debye-Huckel term.

$$V_{DH} = k_{elec} \sum_{i<j} \frac{q_i q_j}{\epsilon_r r_{ij}} e^{-r_{ij}/l_D} \tag{45}$$

where $k_{elec} = (4\pi\epsilon_0)^{-1} = 332.24$kcal Å/mol, $\epsilon_r = 78$, $l_D = 9.6\AA$, q_i and q_j are charges of residue i and j. The distance r_{ij} is the distance between the P atom of DNA residue i and the CB atom of protein residue j. Among protein residues, $q = 1$ for arginine and lysine and $q = -1$ for aspartate and glutamate. the charge of protein residue is assigned to CB atom. The charge of $q = -0.6$ is assigned to the P atom of DNA.

3 Energy validation of the OpenMM implementation of AWSEM, 3SPN.2, and 3SPN.2C

3.1 Energy evaluation comparison with LAMMPS AWSEM

We ran a short simulation of protein phage 434 repressor (PDBID: 1r69) using LAMMPS AWSEM. The structures are saved every 4000 steps. The energies of each energy terms for the first 6 frames evaluated using both OpenMM and LAMMPS implementation are shown here.

	Scheme	Frame	Chain	Chi	Con	Excluded	Rama	Burial	Water	Frag_Mem
0	OpenAWSEM	0	11.22	24.90	18.88	59.50	-236.05	-53.08	-21.00	-331.41
0	LAMMPS	0	11.13	24.99	224.57	59.53	-232.28	-53.08	-21.00	-331.41
1	OpenAWSEM	1	33.69	9.11	63.93	3.52	-281.10	-54.55	-36.98	-323.85
1	LAMMPS	1	33.56	9.14	77.72	3.52	-277.75	-54.55	-36.98	-323.85
2	OpenAWSEM	2	31.17	6.26	46.97	6.91	-288.55	-55.54	-36.49	-327.89
2	LAMMPS	2	31.10	6.28	48.74	6.89	-284.84	-55.54	-36.49	-327.89
3	OpenAWSEM	3	22.94	7.50	46.28	7.82	-293.50	-57.06	-32.85	-327.66
3	LAMMPS	3	22.87	7.53	46.99	7.83	-290.39	-57.06	-32.85	-327.66
4	OpenAWSEM	4	24.39	6.77	51.75	6.68	-284.85	-56.07	-38.37	-324.24
4	LAMMPS	4	24.29	6.79	54.58	6.68	-282.27	-56.07	-38.38	-324.25
5	OpenAWSEM	5	27.66	8.29	47.92	4.34	-286.99	-57.27	-30.29	-325.43
5	LAMMPS	5	27.56	8.29	48.75	4.34	-283.10	-57.27	-30.30	-325.43
6	OpenAWSEM	6	5.41	1.77	0.90	0.85	-300.72	-57.81	-37.07	-331.64
6	LAMMPS	6	5.35	1.78	0.90	0.85	-297.06	-57.81	-37.07	-331.64

Note, the small difference (less than 1 percent) between these two implementations in the multiple terms like the chain term are due the coordination conversion from LAMMPS output format "lammpstrj" to OpenAWSEM format "pdb". In "lammpstrj" format, the positions of atoms are save as the relative position to the simulation box. The difference in the Con term is due a small design change: OpenAWSEM doesn't have the bond between CB and CA for Glycine, but LAMMPS include this bond by using virtual HB as CB.

3.2 Energy evaluation comparison with LAMMPS 3SPN.2 and 3SPN.2C

We ran three short simulations in lammps using the USER-3SPN2 package of a double stranded DNA with sequence ATACAAAGGTGCGAGGTTTCTATGCTCCCACG. The simulations were run for 50000 steps with a timestep of 0.02 ps using the forcefields 3SPN.2 for A-form DNA, B-form DNA and 3SPN.2C respectively. The simulations were ran at a temperature of 300K and a salt concentration of 100mM. A snapshot was taken every 2000 steps.

To make a fair comparison of the implementations in openMM and LAMMPS we recomputed the energies of the resulting 25 frames. We also implemented this comparisons as unit tests in the open3SPN2 software package. The results for the last 6 frames are shown in the tables below.

Forcefield	Frame	Scheme	Angle	Basepair	Bond	CrossStacking	Dihedral	Electrostatics	Exclusion	Stacking	E_{total} (kcal/mol)
ADNA	20	LAMMPS	59.32	-120.75	32.94	-23.62	-157.27	14.14	0.60	-170.80	-365.44
ADNA	20	OpenMM	59.32	-120.75	32.94	-23.62	-157.27	14.14	0.60	-170.80	-365.44
ADNA	21	LAMMPS	55.73	-123.53	28.93	-25.83	-162.05	15.32	0.31	-177.98	-389.10
ADNA	21	OpenMM	55.73	-123.54	28.93	-25.83	-162.05	15.32	0.31	-177.98	-389.10
ADNA	22	LAMMPS	62.99	-125.08	25.42	-23.48	-155.80	14.37	0.93	-170.82	-371.46
ADNA	22	OpenMM	62.99	-125.08	25.42	-23.48	-155.80	14.37	0.93	-170.82	-371.47
ADNA	23	LAMMPS	53.84	-119.08	25.86	-22.89	-158.21	14.69	0.12	-178.91	-384.58
ADNA	23	OpenMM	53.84	-119.08	25.86	-22.89	-158.21	14.69	0.12	-178.91	-384.58
ADNA	24	LAMMPS	52.63	-128.30	21.00	-25.61	-159.19	13.59	0.20	-177.21	-402.90
ADNA	24	OpenMM	52.63	-128.29	21.00	-25.61	-159.19	13.59	0.20	-177.21	-402.90
ADNA	25	LAMMPS	64.75	-123.48	28.13	-26.96	-160.75	14.24	0.18	-169.62	-373.51
ADNA	25	OpenMM	64.75	-123.48	28.13	-26.96	-160.75	14.24	0.18	-169.62	-373.51
BDNA	20	LAMMPS	65.46	-127.77	27.30	-30.59	-155.67	11.75	0.29	-173.75	-382.97
BDNA	20	OpenMM	65.46	-127.77	27.30	-30.59	-155.67	11.75	0.29	-173.75	-382.97
BDNA	21	LAMMPS	49.08	-125.87	26.10	-30.35	-157.98	11.39	0.55	-178.76	-405.84
BDNA	21	OpenMM	49.08	-125.87	26.10	-30.35	-157.98	11.39	0.55	-178.76	-405.85
BDNA	22	LAMMPS	57.63	-131.75	27.05	-29.72	-159.12	11.16	0.05	-176.13	-400.83
BDNA	22	OpenMM	57.63	-131.75	27.05	-29.72	-159.12	11.16	0.05	-176.13	-400.83
BDNA	23	LAMMPS	48.37	-132.69	24.26	-31.00	-156.43	11.40	0.24	-177.33	-413.19
BDNA	23	OpenMM	48.37	-132.69	24.26	-31.00	-156.43	11.40	0.24	-177.33	-413.19
BDNA	24	LAMMPS	54.43	-135.04	25.73	-28.52	-155.46	11.01	0.62	-169.71	-396.96
BDNA	24	OpenMM	54.43	-135.04	25.73	-28.52	-155.46	11.01	0.62	-169.71	-396.96
BDNA	25	LAMMPS	65.59	-128.54	23.00	-29.56	-155.69	11.37	0.73	-167.10	-380.20
BDNA	25	OpenMM	65.59	-128.54	23.00	-29.56	-155.69	11.37	0.73	-167.10	-380.20
B_curved	20	LAMMPS	50.13	-104.57	19.94	-23.45	-181.98	10.24	0.21	-172.50	-401.98
B_curved	20	OpenMM	50.13	-104.57	19.94	-23.45	-181.98	10.24	0.21	-172.50	-401.98
B_curved	21	LAMMPS	64.00	-92.13	27.78	-22.03	-179.08	10.52	0.32	-173.11	-363.73
B_curved	21	OpenMM	64.00	-92.13	27.78	-22.03	-179.08	10.52	0.32	-173.11	-363.73
B_curved	22	LAMMPS	62.70	-108.68	20.72	-25.38	-184.63	10.37	0.27	-171.34	-395.96
B_curved	22	OpenMM	62.70	-108.68	20.72	-25.38	-184.63	10.37	0.27	-171.34	-395.96
B_curved	23	LAMMPS	52.67	-112.61	17.80	-25.01	-188.50	10.12	0.24	-167.98	-413.28
B_curved	23	OpenMM	52.67	-112.61	17.80	-25.01	-188.50	10.12	0.24	-167.98	-413.28
B_curved	24	LAMMPS	60.59	-102.59	20.37	-19.99	-182.96	10.09	1.00	-159.94	-373.44
B_curved	24	OpenMM	60.59	-102.59	20.37	-19.99	-182.96	10.09	1.00	-159.94	-373.44
B_curved	25	LAMMPS	58.18	-99.51	33.32	-19.96	-181.88	10.06	0.25	-163.45	-362.98
B_curved	25	OpenMM	58.18	-99.51	33.33	-19.96	-181.88	10.06	0.25	-163.45	-362.98

4 Tutorial

4.1 open3SPN2

4.1.1 Example DNA system

The following code is also available at `https://github.com/cabb99/open3spn2/tree/master/examples/from_sequence`

```python
# Initialize the DNA from a sequence.
# DNA type can be changed to 'A' or 'B'

seq='ATACAAAGGTGCGAGGTTTCTATGCTCCCACG'
dna=open3SPN2.DNA.fromSequence(seq,dna_type='B_curved')

# Compute the topology for the DNA structure.
# Since the dna was generated from the sequence using X3DNA,
# it is not necesary to recompute the geometry.

dna.computeTopology(template_from_X3DNA=False)

# Create the system.
# To set periodic boundary conditions (periodicBox=[50,50,50]).
# The periodic box size is in nanometers.
dna.periodic=False
s=open3SPN2.System(dna, periodicBox=None)

#Add 3SPN2 forces
s.add3SPN2forces(verbose=True)

import simtk.openmm
import simtk.openmm.app
import simtk.unit
import sys
import numpy as np

```

```python
#Initialize Molecular Dynamics simulations
s.initializeMD(temperature=300 * simtk.unit.kelvin,platform_name='OpenCL')
simulation=s.simulation

#Set initial positions
simulation.context.setPositions(s.coord.getPositions())

energy_unit=simtk.openmm.unit.kilojoule_per_mole
#Total energy
state = simulation.context.getState(getEnergy=True)
energy = state.getPotentialEnergy().value_in_unit(energy_unit)
print('TotalEnergy',round(energy,6),energy_unit.get_symbol())

#Detailed energy
energies = {}
for force_name, force in s.forces.items():
    group=force.getForceGroup()
    state = simulation.context.getState(getEnergy=True, groups=2**group)
    energies[force_name] =state.getPotentialEnergy().value_in_unit(energy_unit)

for force_name in s.forces.keys():
    print(force_name, round(energies[force_name],6),energy_unit.get_symbol())

    #Add simulation reporters
dcd_reporter=simtk.openmm.app.DCDReporter(f'output.dcd', 1000)
energy_reporter=simtk.openmm.app.StateDataReporter(sys.stdout, 1000, step=True,time=True,
                potentialEnergy=True, temperature=True)
simulation.reporters.append(dcd_reporter)
simulation.reporters.append(energy_reporter)

#Run simulation
simulation.step(10000)

```

4.1.2 Example Protein-DNA system

The following code is also available at `https://github.com/cabb99/open3spn2/tree/master/examples/Protein_DNA`

```python
# If you want to specify the package address
# you can add them to the PYTHONPATH environment variable.
# Also you can add them on the run time uncommenting the lines below
# import sys
# open3SPN2_HOME = '/Users/weilu/open3spn2/'
# openAWSEM_HOME = '/Users/weilu/openmmawsem/'
# sys.path.insert(0,open3SPN2_HOME)
# sys.path.insert(0,openAWSEM_HOME)

#Import openAWSEM, open3SPN2 and other libraries
import open3SPN2
import ffAWSEM
import pandas
import numpy as np
import simtk.openmm
from functools import partial
```

```python
import sys

#Fix the system (adds missing atoms)
fix=open3SPN2.fixPDB("1lmb.pdb")

#Create a table containing both the proteins and the DNA
complex_table=open3SPN2.pdb2table(fix)

# Create a single memory file
ffAWSEM.create_single_memory(fix)

#Generate a coarse-grained model of the DNA molecules
dna_atoms=open3SPN2.DNA.CoarseGrain(complex_table)

#Generate a coarse-grained model of the Protein molecules
protein_atoms=ffAWSEM.Protein.CoarseGrain(complex_table)

#Merge the models
Coarse=pandas.concat([protein_atoms,dna_atoms],sort=False)
Coarse.index=range(len(Coarse))
Coarse['serial']=list(Coarse.index)

#Save the protein_sequence
ffAWSEM.save_protein_sequence(Coarse,sequence_file='protein.seq')

# Create a merged PDB
ffAWSEM.writePDB(Coarse,'clean.pdb')

#Create the merged system
pdb=simtk.openmm.app.PDBFile('clean.pdb')
top=pdb.topology
coord=pdb.positions
forcefield=simtk.openmm.app.ForceField(ffAWSEM.xml,open3SPN2.xml)
s=forcefield.createSystem(top)

#Create the DNA and Protein Objects
dna=open3SPN2.DNA.fromCoarsePDB('clean.pdb')
with open('protein.seq') as ps:
    protein_seq=ps.readlines()[0]
protein=ffAWSEM.Protein.fromCoarsePDB('clean.pdb',
                                      sequence=protein_seq)
dna.periodic=False
protein.periodic=False

#Copy the AWSEM parameter files
ffAWSEM.copy_parameter_files()

#Clear Forces from the system (optional)
keepCMMotionRemover=True
j=0
for i, f in enumerate(s.getForces()):
    if keepCMMotionRemover and i == 0 and f.__class__ == simtk.
    openmm.CMMotionRemover:
        # print('Kept ', f.__class__)
        j += 1
        continue
    else:
        # print('Removed ', f.__class__)
        s.removeForce(j)
if keepCMMotionRemover == False:
    assert len(s.getForces()) == 0, 'Not all the forces were
    removed'
```

```python
else:
    assert len(s.getForces()) <= 1, 'Not all the forces were removed'

#Initialize the force dictionary
forces={}
for i in range(s.getNumForces()):
    force = s.getForce(i)
    force_name="CMMotionRemover"

#Add 3SPN.2 forces
for force_name in open3SPN2.forces:
    print(force_name)
    force = open3SPN2.forces[force_name](dna)
    if force_name in ['BasePair','CrossStacking']:
        force.addForce(s)
    else:
        s.addForce(force)
    forces.update({force_name:force})

#Add AWSEM forces
ft=ffAWSEM.functionTerms
openAWSEMforces = dict(Connectivity=ft.basicTerms.con_term,
                       Chain=ft.basicTerms.chain_term,
                       Chi=ft.basicTerms.chi_term,
                       Excl=ft.basicTerms.excl_term,
                       rama=ft.basicTerms.rama_term,
                       rama_pro=ft.basicTerms.rama_proline_term,
                       contact=ft.contactTerms.contact_term,
                       frag  = partial(ft.templateTerms.fragment_memory_term,
                                       frag_file_list_file = "./single_frags.mem",
                                       npy_frag_table = "./single_frags.npy",
                                       UseSavedFragTable = False,
                                       k_fm = 0.04184/3),
                       beta1 = ft.hydrogenBondTerms.beta_term_1,
                       beta2 = ft.hydrogenBondTerms.beta_term_2,
                       beta3 = ft.hydrogenBondTerms.beta_term_3,
                       pap1 = ft.hydrogenBondTerms.pap_term_1,
                       pap2 = ft.hydrogenBondTerms.pap_term_2,
                      )
protein.setup_virtual_sites(s)

#Add DNA-protein interaction forces
for force_name in open3SPN2.protein_dna_forces:
    print(force_name)
    force = open3SPN2.protein_dna_forces[force_name](dna,protein)
    s.addForce(force)
    forces.update({force_name: force})

#Fix exclussions
for force_name in openAWSEMforces:
    print(force_name)
    if force_name in ['contact']:
        force = openAWSEMforces[force_name](protein,
                                            withExclusion=False,
                                            periodic=False)
        print(force.getNumExclusions())
        open3SPN2.addNonBondedExclusions(dna,force)
        print(force.getNumExclusions())
```

```
    elif force_name in ['Excl']:
        force = openAWSEMforces[force_name](protein)
        print(force.getNumExclusions())
        open3SPN2.addNonBondedExclusions(dna,force)
        print(force.getNumExclusions())
    else:
        force = openAWSEMforces[force_name](protein)
    s.addForce(force)
    forces.update({force_name: force})

#Initialize the simulation
temperature=300 * simtk.openmm.unit.kelvin
platform_name='OpenCL' #'Reference','CPU','CUDA', 'OpenCL'
integrator = simtk.openmm.LangevinIntegrator(temperature,
               1 / simtk.openmm.unit.picosecond,
               2 * simtk.openmm.unit.femtoseconds)
platform = simtk.openmm.Platform.getPlatformByName(platform_name)
simulation = simtk.openmm.app.Simulation(top,s, integrator, platform)
simulation.context.setPositions(coord)
energy_unit=simtk.openmm.unit.kilojoule_per_mole
state = simulation.context.getState(getEnergy=True)
energy = state.getPotentialEnergy().value_in_unit(energy_unit)
print(energy)

#Obtain total energy
energy_unit=simtk.openmm.unit.kilojoule_per_mole
state = simulation.context.getState(getEnergy=True)
energy = state.getPotentialEnergy().value_in_unit(energy_unit)
print('TotalEnergy',round(energy,6),energy_unit.get_symbol())

#Obtain detailed energy
energies = {}
for force_name, force in forces.items():
    group=force.getForceGroup()
    state = simulation.context.getState(getEnergy=True,
                                        groups=2**group)
    energies[force_name] =state.getPotentialEnergy().value_in_unit(energy_unit)

for force_name in forces.keys():
    print(force_name, round(energies[force_name],6),
          energy_unit.get_symbol())

#Add simulation reporters
dcd_reporter=simtk.openmm.app.DCDReporter(f'output.dcd', 10000)
energy_reporter=simtk.openmm.app.StateDataReporter(sys.stdout, 10000, step=True,time=True, potentialEnergy=True, temperature=True)
simulation.reporters.append(dcd_reporter)
simulation.reporters.append(energy_reporter)

#Run simulation
simulation.minimizeEnergy()
simulation.context.setVelocitiesToTemperature(temperature)
simulation.step(100000)
```

5 Supplementary figures

5.1 Structure prediction results using three contact potential schemes evaluated using the overall Q

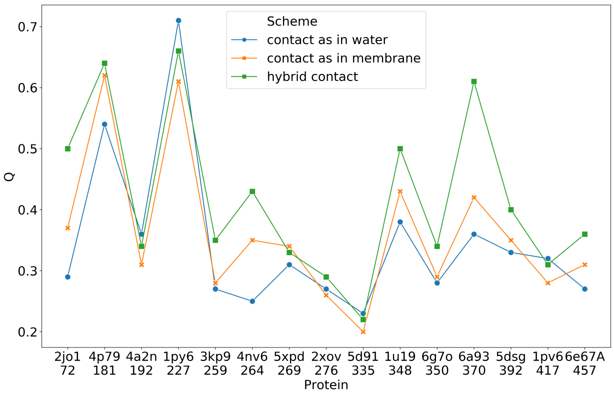

Figure 9: **Structure prediction results using three contact potential schemes evaluated using the overall Q.**

5.2 Example of over saturation of disulfide bonds observed in original AWSEM simulation.

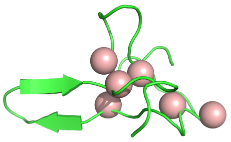

Figure 10: **Example of over saturation of disulfide bonds observed in original AWSEM simulation.** One cystine is in contact with three other cystines.

5.3 Bets Q for each run.

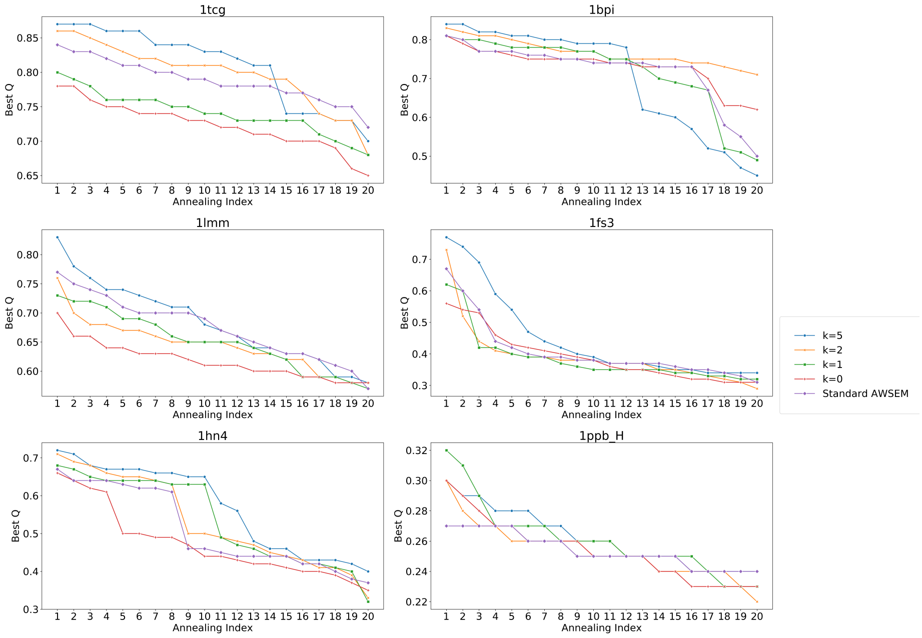

Figure 11: **Best Q value for each run.** a large strength of the disulfide bond potential leads to higher Q value. The annealing indexes are given by sorting their Q value from high to low.

5.4 The predicted structure of alpha-thrombin(PDB: 1ppb) aligned with the crystal structure.

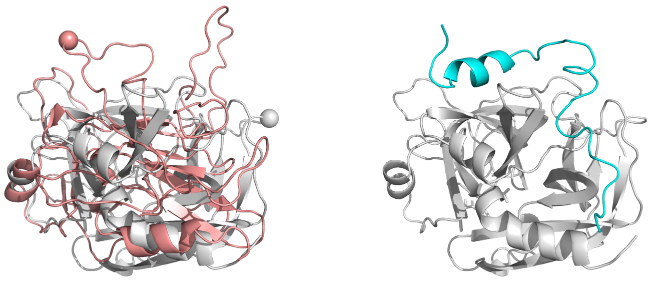

Figure 12: **Left: The structure alignment of predicted structure(red) and crystal structure(white). Right: The complete thrombin crystal structure.** Overall,the lower left region(the C terminal region; residue 168-259) is well aligned. But there is a partial mirror image shown in upper right, residue 150 is shown as sphere as an indication of the mirror image. This partial native folding might due to that we didn't model the short chain that is experimentally proven to be important for thrombin function [5].

5.5 The predicted structure of ribonuclease A(PDB: 1fs3) aligned with the crystal structure.

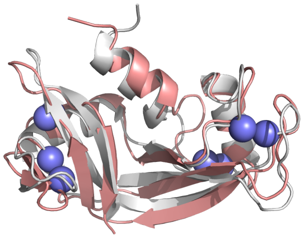

Figure 13: **The structure alignment of predicted structure(red) and crystal structure(white).** The sphere is the CB of Cystines. All Cystine pairs in the predicted structure is matched with the Cystine pairs in crystal structure.

References

[1] Hinckley DM, Freeman GS, Whitmer JK, De Pablo JJ. An experimentally-informed coarse-grained 3-site-per-nucleotide model of DNA: Structure, thermodynamics, and dynamics of hybridization. Journal of Chemical Physics. 2013;139(14). doi:10.1063/1.4822042.

[2] Freeman GS, Hinckley DM, Lequieu JP, Whitmer JK, de Pablo JJ. Coarse-grained modeling of DNA curvature. The Journal of chemical physics. 2014;141(16):165103. doi:10.1063/1.4897649.

[3] Lu XJ, Olson WK. 3DNA: a software package for the analysis, rebuilding and visualization of three-dimensional nucleic acid structures. Nucleic acids research. 2003;31(17):5108–5121.

[4] Zhang B, Zheng W, Papoian GA, Wolynes PG. Exploring the free energy landscape of nucleosomes. Journal of the American Chemical Society. 2016;138(26):8126–8133.

[5] Papaconstantinou M, Bah A, Di Cera E. Role of the A chain in thrombin function. Cellular and Molecular Life Sciences. 2008;65(12):1943–1947.
