# Supplementary material for: OpenAWSEM with Open3SPN2: A fast, flexible, and accessible framework for large-scale coarse-grained biomolecular simulations
Source: PLoS Comput Biol. 2021 Feb 12;17(2):e1008308. doi: 10.1371/journal.pcbi.1008308 (PMC7906472; doi:10.1371/journal.pcbi.1008308)
Supplement: Attachment — Submitted filename: response_letter_dec19.pdf [file pcbi.1008308.s002.pdf]

Dear Editor and Reviewers –

After our manuscript was sent for review, we were made aware by a colleague that the LAMMPS implementation of AWSEM had recently been restructured and its computational efficiency improved. We feel that the manuscript will benefit the community most if we compare OpenAWSEM to the latest version of the LAMMPS implementation of AWSEM. We have carried out new benchmark simulations using the updated LAMMPS implementation of AWSEM. We have updated the benchmark figures (Figures 1 and 3) and the text of the manuscript accordingly. Finally, in recognition of his contributions to the updated manuscript, we have added our colleague Aram Davtyan as a co-author on the manuscript.

The sentence,

> *"By utilizing GPUs, we achieve more than a 100-fold speedup in protein and protein-DNA simulations over the existing LAMMPS-based implementations running on a CPU."*

has been modified to read,

> *"By utilizing GPUs, we achieve around a 30-fold speedup in protein and protein-DNA simulations over the existing LAMMPS-based implementations running on a single CPU core."*

Also, the sentence,

> *"For a protein with 1716 residues (PDB 6n7n), a simulation of 1 million steps corresponding roughly to 5$\mu s$ in laboratory time took more than 200 hours (8 days) using LAMMPS. The same simulation takes only about 1 hour using OpenAWSEM, thus making millisecond simulations feasible within a few days."*

now reads,

> *"For a protein with 3724 residues (PDB 4qqw), a simulation of 4 million steps corresponding roughly to 20$\mu s$ in laboratory time took more than 200 hours (8 days) using LAMMPS. The same simulation takes only about 8 hours using OpenAWSEM, thus making millisecond simulations feasible within a few days."*

Finally, where previously we said,

> *"In this new framework, simulations using GPUs can achieve speedups of a factor of a hundred for the simulation of proteins that have more than one thousand residues."*

the manuscript now reads,

> *"In this new framework, simulations using GPUs can achieve speedups of a factor of thirty for the simulation of proteins that have more than two thousand residues."*

Reviewer #1: Authors present an implementation of the AWSEM force-field for proteins and the 3SPN.2 model for DNA to openMM, together with two new potential energy functions. By the implementation and the use of GPU, protein simulations with AWSEM speed up two orders of magnitude, which is very impressive. In addition, taking advantage of the extensibility of openMM, authors implemented two non-trivial energy functions; one for the hybrid use of water- and membrane- environmental contact energies, and the other for many-body covalent bond potential. Both of the new potentials were shown to improve the simulations significantly.

Together, I consider this a very impressive and useful software development and thus I recommend its publication after addressing the following minor points.

We appreciate the reviewer's helpful comments and positive appraisal of our work.

1) The speed up by openMM/GPU relative to LAMMPS/CPU reaches two order of magnitude for AWSEM, but is limited to less than 10 for 3SPN.2. Also, for a short DNA sequence, the openMM version is slower. Authors should provide some reasonings of this difference, if possible. At least, some discussions must be possible.

The greater improvement in efficiency is mainly due to the presence of the density-dependent water-mediated term in the AWSEM forcefield. The water-mediated term is the most computationally-intensive term in AWSEM as its evaluation involves the computation of both pairwise distances between pairs of residues and the local densities for each residue. For short sequences, the GPU is underutilized and the greater overhead associated with using the GPU results in longer overall simulation times.

In main text, we have added the following remark into the section on DNA-only simulations:

> *"For short sequences, the GPU is underutilized and the greater overhead associated with using the GPU results in longer overall simulation times."*

2) Related to the above point, can author describe the dominant part of the calculations with openMM/GPU? Is it contact potential for AWSEM? For 3SPN.2, the bottleneck may be the electrostatic interaction.

Yes, most of the computation time is used on the density-dependent contact potentials in AWSEM. For 3SPN.2, the heaviest computations are the CrossStacking interactions, which involve the positions of 5 atoms.

3) I like the many-body potential function of disulfide bonds. Yet, I am not aware of the motivation to use theta^near function. Does this increase the specificity? Is it really necessary ?

Yes, it does increase the specificity. We added the theta^near term so that when one unpaired cysteine comes near to an already paired cysteine the two cysteines (one paired and one unpaired) will not be attracted to each other.

4) A very minor point. In the section: Availability and Future Directions, I do not find any comments on future directions.

Future directions could include studying protein-protein interactions such as the dimerization or oligomerization of membrane proteins, as mentioned by another reviewer.

In the Future Directions section we added the following remark:

> *"We plan to study protein-protein interactions such as the dimerization or oligomerization of membrane protein in the future."*

Reviewer #2: The authors implemented existing coarse-grained models for protein (AWSEM) and DNA (3SPN2) under the OpenMM framework. The new implementation enabled MD simulations of protein and/or DNA using GPUs, and the simulation speeds were accelerated dramatically as compared to the implementation in LAMMPS, especially for large proteins. In addition, two new potentials were also proposed: one for the interaction between protein and membrane, the other for proteins with multiple disulfide bonds to avoid unphysical clustering of cysteines. The predicted structures were in good agreement with experimental ones in the applications to protein-DNA complexes, proteins partially buried in a membrane and proteins with multiple disulfide bonds. The software will be useful to the community of molecular biophysics.

We appreciate the reviewer's helpful comments and positive appraisal of our work.

Here comes the questions and concerns:
1. In the simulation of the protein-DNA complex, the protein and DNA were first pulled apart. How far apart are the protein and DNA?

They are pulled 100 Å away from each other.

We changed the phrase,

> *"the protein and DNA in the crystal structure were first pulled apart and run for 2.5 million steps"*

to

> *"the protein and DNA in the crystal structure were first pulled 100Å apart and run for 2.5 million steps"*

2. Following the instruction in github, the tutorial for DNA simulation with open3SPN2 can be successfully completed. However, the tutorial for protein-DNA simulation with open3SPN2

cannot be completed and failed with errors. Could the author double check the code of the tutorial (both the one in the SI and the one online)?

We checked the code for the tutorial and it has been updated both in the manuscripts as well as in the open3SPN2 documentation page. We appreciate the reviewer's efforts in testing the tutorial.

3. With the two new potentials, could the authors comment on the potential applications other than the prediction of protein native structures? How about the simulation of dimerization or oligomerization of membrane proteins? How about the potential kinetics and dynamics studies?

Yes. Similar to the core AWSEM forcefield, the new potentials can be used to study dimerization or oligomerization of membrane proteins or for doing studies of dynamics.

4. To facilitate the wide application of the software, a tutorial on how to simulate proteins partially buried in membrane will be helpful.

Thanks for the suggestion. We have made a tutorial on this. Please checkout https://github.com/npschafer/openawsem/wiki/Tutorial-on-simulating-a-membrane-protein-with-globular-part

5. At the end of page 3 in SI, "The parameters are defined in 1" should be "The parameters are defined in Table 1"

Thanks. We have changed the text accordingly.

6. On page 9 of SI, the table number is not shown properly.

Thanks. It has been corrected.

---------------

[1] A letter containing a detailed list of your responses to all review comments, and a description of the changes you have made in the manuscript. Please note while forming your response, if your article is accepted, you may have the opportunity to make the peer review history publicly available. The record will include editor decision letters (with reviews) and your responses to reviewer comments. If eligible, we will contact you to opt in or out

[2] Two versions of the revised manuscript: one with either highlights or tracked changes denoting where the text has been changed; the other a clean version (uploaded as the manuscript file).
